# (Croconato-κ^2^ *O*,*O*′)bis­(1,10-phenanthroline-κ^2^ *N*,*N*′)zinc(II)

**DOI:** 10.1107/S1600536808033709

**Published:** 2008-11-08

**Authors:** Hongyu Chen, Ping Li, Lihua Dong, Xiaohui Zhu, Qi Fang

**Affiliations:** a School of Chemistry and Chemical Engineering, TaiShan Medical University, Tai’an 271016, People’s Republic of China; b State Key Laboratory of Crystal Materials, Shandong University, Jinan 250100, Shandong Province, People’s Republic of China

## Abstract

In the title compound, [Zn(C_5_O_5_)(C_12_H_8_N_2_)_2_], the Zn atom is in a slightly distorted octa­hedral environment. The mol­ecule lies across a twofold rotation axis, around which two 1,10-phenanthroline ligands are arranged. There are short contacts between the 1,10-phenanthroline groups and the O atoms of the croconate ligand, which probably stabilize the crystal structure *via* weak C—H⋯O interactions.

## Related literature

For related literature, see: Braga *et al.* (2002 ▶); Carranza *et al.* (2004 ▶); Castro *et al.* (1992 ▶, 2002 ▶); Chen *et al.* (2005 ▶, 2007 ▶, 2008 ▶); Faus *et al.* (1994 ▶); Maji *et al.* (2003 ▶); Seitz & Imming (1992 ▶); Sletten *et al.* (1998 ▶); Wang *et al.* (2002 ▶). 
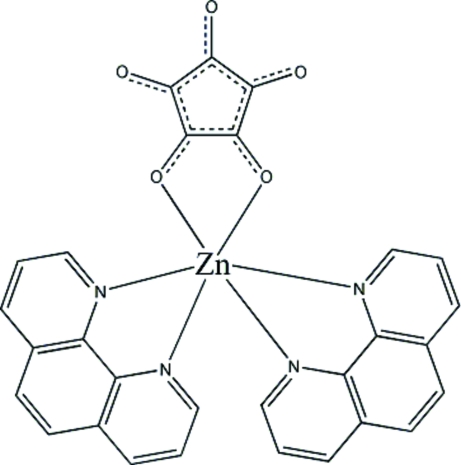


## Experimental

### 

#### Crystal data

[Zn(C_5_O_5_)(C_12_H_8_N_2_)_2_]
*M* *_r_* = 565.83
Orthorhombic, 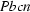

*a* = 12.2605 (4) Å
*b* = 11.0133 (3) Å
*c* = 17.2745 (5) Å
*V* = 2332.55 (12) Å^3^
*Z* = 4
Mo *K*α radiation
μ = 1.11 mm^−1^
*T* = 293 (2) K
0.28 × 0.23 × 0.15 mm

#### Data collection

Bruker APEXII CCD area-detector diffractometer
Absorption correction: multi-scan (*APEX2*; Bruker, 2005 ▶) *T*_min_ = 0.743, *T*_max_ = 0.782 (expected range = 0.805–0.847)
9769 measured reflections
2627 independent reflections
2164 reflections with *I* > 2σ(*I*)
*R*_int_ = 0.019

#### Refinement

*R*[*F* ^2^ > 2σ(*F* ^2^)] = 0.029
*wR*(*F* ^2^) = 0.096
*S* = 1.28
2627 reflections
179 parameters
H-atom parameters constrained
Δρ_max_ = 0.29 e Å^−3^
Δρ_min_ = −0.32 e Å^−3^

### 

Data collection: *APEX2* (Bruker, 2005 ▶); cell refinement: *APEX2*; data reduction: *APEX2*; program(s) used to solve structure: *SIR97* (Altomare *et al.*, 1999 ▶); program(s) used to refine structure: *SHELXL97* (Sheldrick, 2008 ▶); molecular graphics: *SHELXTL* (Sheldrick, 2008 ▶); software used to prepare material for publication: *WinGX* (Farrugia, 1999 ▶).

## Supplementary Material

Crystal structure: contains datablocks global, I. DOI: 10.1107/S1600536808033709/sg2254sup1.cif

Structure factors: contains datablocks I. DOI: 10.1107/S1600536808033709/sg2254Isup2.hkl

Additional supplementary materials:  crystallographic information; 3D view; checkCIF report

## Figures and Tables

**Table 1:** Selected bond lengths (Å)

N1—Zn1	2.1493 (15)
N2—Zn1	2.1664 (17)
O1—Zn1	2.1325 (14)

**Table 2:** Hydrogen-bond geometry (Å, °)

*D*—H⋯*A*	*D*—H	H⋯*A*	*D*⋯*A*	*D*—H⋯*A*
C6—H6⋯O1^i^	0.93	2.47	3.295 (2)	149
C11—H11⋯O3^ii^	0.93	2.55	3.147 (2)	122

Symmetry codes: (i) 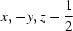; (ii) 
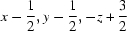
.

**Fig. 1.**
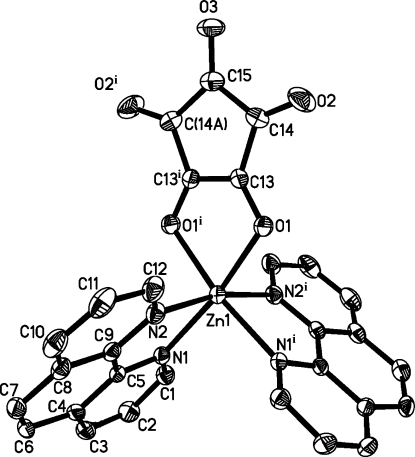
Molecular structure with thermal ellipsoids at 30% probability levels. [symmetry code: -x + 1/2, y, -z + 3/2]

**Fig. 2.**
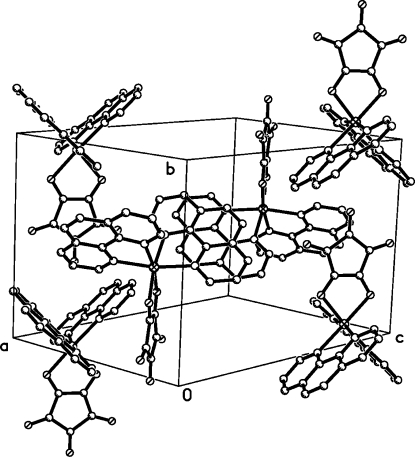
A packing diagram of the title compound.

## supplementary crystallographic information

### Comment

The dianion of croconic acid(4,5-dihydroxycyclopent-4-ene-1,2,3-trione), (C_5_O_5_)^2-^, is one of the cyclic aromatic oxocarbons (CO)_n_^2-^ characterized by extensive delocalization of the π electrons all over the ring (Seitz & Imming, 1992). Previous reports reveal that croconate is a polydent ligand(Chen *et al.*, 2005, Maji *et al.*, 2003, Wang *et al.*, 2002, Sletten *et al.*, 1998). According to the concept of 'molecular self-organization' and 'molecular engineering', transition metal croconates associated with another ligand such as terpyridine (Castro *et al.* 2002), bis((bis(2-pyridylcarbonyl) amido (Maji *et al.*, 2003), 2,2'-bipyridine (Castro *et al.*, 1992) or the tetrakis(2-pyridyl)pyrazine (Carranza *et al.* 2004) show interesting properties in magnetism, biochemistry, catalyst *et al.*

1,10-Phenanthroline(phen) is a well known neutral bidentate ligand. There have been considerable interests in the synthesis of open-framework phen-based metal complexes because of their interesting structure chemistry and potential applications(Faus *et al.* 1994). Recently, many research activities have focused on the synthesis of hybrid framework by incorporate organic ligand in the structure of phen-based complexes. Among the family of cyclic oxocarbons of the formula (CO)_n_^2-^ [n=2–6 for oxalate, deltate, squarate, croconate and rhodizonate anions, respectively], the croconate moiety, (C_5_O_5_)^2-^, was found to be a good candidate and has been successfully incorporated into phen-based frameworks in our previous work, [*M*(phen)_2_(C_5_O_5_)] (*M*= Cu, Ni, Co, Mn) (Chen *et al.*, 2005, 2007, 2008). Here, we report a new member of this family: [Zn(phen)_2_(C_5_O_5_)].

In the title structure, asymmetry unit contains a phen moiety and half a coroconte group coordinated with a zinc ion. The title compound lies across twofold rotation axes which passes through the Zn atom and bisects the croconate ligand, around which two phen ligand are arranged in a chiral propeller manner. A unit cell contains four [Zn(phen)_2_(C_5_O_5_)] molecules.

As a good π-conjugation system, the croconate dianion('free' ligand) in its simple salt has a plannar D_5 h_ conformation with five almost identical C?O bonds and five almost identical C?C bonds, such as in Rb_2_C_5_O_5_ and Cs_2_C_5_O_5_ crystals (Braga *et al.*, 2002). However, the coordinated ligand in title complex obvioulsy deviates from D_5 h_ symmetry. The C?O bond involving coordinated O atoms is longer than that involving the uncoordinated O atoms. In the title complex, the C?O bond lengths are 1.229 (3) Å and 1.228 (4) Å for the uncoordinated O atoms and 1.277 (2) Å for the coordinated O atoms.

The molecuar conformation of [Zn(phen)_2_(C_5_O_5_)] is close to [Co(phen)_2_(C_5_O_5_)] and [Ni(phen)_2_(C_5_O_5_)] while different from [Cu(phen)_2_(C_5_O_5_)] and [Mn(phen)_2_(C_5_O_5_)]. The dihedral angle between the two phen planes for the title compound is 85.3 (1)° and the croconate and phen planes are also effectively perpendicular, with a dihedral angle of 87.7 (1)°. Compared with our previous reported result(Chen *et al.* 2005, 2007, 2008), the crystal growth method not only influence crystal packing motif, but also have effect on the moleular configuration. The crystal of [Zn(phen)_2_(C_5_O_5_)], [Co(phen)_2_(C_5_O_5_)] and [Ni(phen)_2_(C_5_O_5_)] are grown by hydrothermal method while the cyrstal of [Cu(phen)_2_(C_5_O_5_)] and [Mn(phen)_2_(C_5_O_5_)] are obtained by solvent evaporation under room temperature. Correspondingly, the crystal packing motif for crystal of Ni, Co, and Zn complexes are P_bcn_, while it is *C*2/*c* for Cu and Mn complexes. As for the molecular configuration, the dihendral angles between the two phen planes for the complexes of Zn, Co, Ni are 87.7 (1)°, 85.7 (1)°, 86.0 (1)° which are almost perpendicular to each other, but they are 46.5 (1)° and 40.7 (1)° for Cu and Mn complexes.

According to the Jahn-teller effect theotry (if a d-orbital of a transiton metal ion is empty or full-filled and the other equivalent orbital is half full-filled, the coordinate enviorment of the transition metal ion will be distorted to form a more stable configuration), Cu^2+^ and Mn^2+^ have a strong tendency to Jahn-teller distortion while Zn^2+^ and Ni^2+^ are not. So, the local polyhedral MN_4_O_2_(*M*=Zn, Co, Ni) in their complexes are close to the octahedral while MN_4_O_2_(*M*=Mn, Cui) in their complexes are severely distorted from the octahedral. In the tiltle comound, the values of the angles subtended by the bidentate at zinc atom [77.37 (6)°] deviate significantly from the ideal vaue of 90° due to the small bite size of the five-memberbered plannar chelate rings.

As shown in Fig.2, the dipole moments of [Zn(phen)_2_(C_5_O_5_)] are arranged alternatively along +b and-b directions. There are short contacts between the phen groups and the O atoms of the croconate [*e.g*. C(6)—H(6)···O(1) (*x*, -*y*, -1/2 + *z*) and C(11)—H(11)···O(3)(-1/2 + *x*, -1/2 + *y*, 3/2 - *z*), which probably stablize the crystal structure.

### Experimental

[K_2_(C_5_O_5_)](0.10 g) and Zn(CH_3_COO)_2_.2H_2_O (0.10 g) were dissolved in solvent of water 15 ml. The mixture was heated to 340–350 K under continuous stirring for 20 min. To the resulting yellow solution, an ethanol solution (10 ml) of 1,10-phenanthroline (0.1 mol/*L*) was added to cause an immediate precipation of yellow microcrystal. After the solutions were left to stand at room temperature for 30 minutes, they were collected by filter suction, washed with water. Then the obtained precipation and 15 ml water was placed in the teflon liner of an autoclave, which was sealed and heated to 433 K for 48 h, cooled at speed of 10 K/min, whereupon yellow block of [Zn(phen)_2_(C_5_O_5_)] were obtained.

### Refinement

All H atoms were geometrically fixed and allowed to ride on their attached atoms, which C—H = 0.93 Å and *U*_iso_(H)= 1.2 *U*_eq_(C).

### Crystal data

**Table:**

[Zn(C_5_O_5_)(C_12_H_8_N_2_)_2_]	*F*_000_ = 1152
*M**_r_* = 565.83	*D*_x_ = 1.611 Mg m^−^^3^
Orthorhombic, *P**b**c**n*	Mo *K*α radiation λ = 0.71073 Å
Hall symbol: -P 2n 2ab	Cell parameters from 3967 reflections
*a* = 12.2605 (4) Å	θ = 2.8–27.5º
*b* = 11.0133 (3) Å	µ = 1.11 mm^−^^1^
*c* = 17.2745 (5) Å	*T* = 293 (2) K
*V* = 2332.55 (12) Å^3^	Prism, yellow
*Z* = 4	0.28 × 0.23 × 0.15 mm

### Data collection

**Table:**

Bruker APEXII CCD area-detector diffractometer	2627 independent reflections
Radiation source: fine-focus sealed tube	2164 reflections with *I* > 2σ(*I*)
Monochromator: graphite	*R*_int_ = 0.019
Detector resolution: 10.0 pixels mm^-1^	θ_max_ = 27.6º
*T* = 295(2) K	θ_min_ = 2.4º
φ and ω scans	*h* = −15→14
Absorption correction: multi-scan(APEX2; Bruker, 2005)	*k* = −14→13
*T*_min_ = 0.743, *T*_max_ = 0.782	*l* = −21→21
9769 measured reflections	

### Refinement

**Table:**

Refinement on *F*^2^	Hydrogen site location: inferred from neighbouring sites
Least-squares matrix: full	H-atom parameters constrained
*R*[*F*^2^ > 2σ(*F*^2^)] = 0.029	*w* = 1/[σ^2^(*F*_o_^2^) + (0.0414*P*)^2^ + 0.6435*P*] where *P* = (*F*_o_^2^ + 2*F*_c_^2^)/3
*wR*(*F*^2^) = 0.096	(Δ/σ)_max_ < 0.001
*S* = 1.28	Δρ_max_ = 0.29 e Å^−^^3^
2627 reflections	Δρ_min_ = −0.32 e Å^−^^3^
179 parameters	Extinction correction: SHELXL97 (Sheldrick, 2008), Fc^*^=kFc[1+0.001xFc^2^λ^3^/sin(2θ)]^-1/4^
Primary atom site location: structure-invariant direct methods	Extinction coefficient: 0.0037 (5)
Secondary atom site location: difference Fourier map	

### Special details

**Table:**

Geometry. All e.s.d.'s (except the e.s.d. in the dihedral angle between two l.s. planes) are estimated using the full covariance matrix. The cell e.s.d.'s are taken into account individually in the estimation of e.s.d.'s in distances, angles and torsion angles; correlations between e.s.d.'s in cell parameters are only used when they are defined by crystal symmetry. An approximate (isotropic) treatment of cell e.s.d.'s is used for estimating e.s.d.'s involving l.s. planes.
Refinement. Refinement of *F*^2^ against ALL reflections. The weighted *R*-factor *wR* and goodness of fit *S* are based on *F*^2^, conventional *R*-factors *R* are based on *F*, with *F* set to zero for negative *F*^2^. The threshold expression of *F*^2^ > σ(*F*^2^) is used only for calculating *R*-factors(gt) *etc*. and is not relevant to the choice of reflections for refinement. *R*-factors based on *F*^2^ are statistically about twice as large as those based on *F*, and *R*- factors based on ALL data will be even larger.

### Fractional atomic coordinates and isotropic or equivalent isotropic displacement parameters (Å^2^)

**Table:**

	*x*	*y*	*z*	*U*_iso_*/*U*_eq_	
C1	1.14583 (16)	−0.08987 (18)	0.66696 (12)	0.0370 (4)	
H1	1.1977	−0.0725	0.7047	0.044*	
C2	1.17077 (19)	−0.1768 (2)	0.61125 (13)	0.0442 (5)	
H2	1.2374	−0.2171	0.6123	0.053*	
C3	1.09642 (18)	−0.20164 (18)	0.55543 (12)	0.0411 (5)	
H3	1.1121	−0.2598	0.5180	0.049*	
C4	0.99594 (16)	−0.14043 (18)	0.55356 (12)	0.0346 (4)	
C5	0.97663 (15)	−0.05585 (16)	0.61341 (10)	0.0284 (4)	
C6	0.91524 (18)	−0.15647 (19)	0.49514 (12)	0.0406 (5)	
H6	0.9282	−0.2110	0.4551	0.049*	
C7	0.82034 (19)	−0.09419 (19)	0.49667 (13)	0.0434 (5)	
H7	0.7700	−0.1051	0.4570	0.052*	
C8	0.79598 (16)	−0.01181 (19)	0.55831 (11)	0.0371 (4)	
C9	0.87469 (15)	0.00774 (16)	0.61615 (11)	0.0305 (4)	
C10	0.69824 (18)	0.0534 (2)	0.56380 (14)	0.0503 (6)	
H10	0.6443	0.0438	0.5264	0.060*	
C11	0.68264 (19)	0.1309 (2)	0.62399 (15)	0.0535 (6)	
H11	0.6177	0.1740	0.6281	0.064*	
C12	0.76426 (18)	0.1457 (2)	0.67964 (13)	0.0460 (5)	
H12	0.7522	0.1985	0.7208	0.055*	
C13	0.96697 (16)	0.35065 (18)	0.78378 (11)	0.0334 (4)	
C14	0.94811 (17)	0.47521 (18)	0.80990 (13)	0.0409 (5)	
C15	1.0000	0.5549 (3)	0.7500	0.0419 (7)	
N1	1.05186 (12)	−0.03074 (13)	0.66885 (9)	0.0290 (3)	
N2	0.85892 (13)	0.08653 (15)	0.67547 (9)	0.0352 (4)	
O1	0.93325 (12)	0.25245 (13)	0.81491 (8)	0.0415 (3)	
O2	0.89952 (16)	0.50881 (16)	0.86832 (11)	0.0659 (5)	
O3	1.0000	0.6664 (2)	0.7500	0.0560 (7)	
Zn1	1.0000	0.10546 (3)	0.7500	0.03197 (13)	

### Atomic displacement parameters (Å^2^)

**Table:**

	*U*^11^	*U*^22^	*U*^33^	*U*^12^	*U*^13^	*U*^23^
C1	0.0350 (10)	0.0405 (11)	0.0355 (11)	0.0036 (8)	−0.0035 (8)	0.0000 (8)
C2	0.0441 (11)	0.0421 (11)	0.0465 (13)	0.0118 (9)	0.0055 (10)	−0.0014 (9)
C3	0.0551 (13)	0.0326 (10)	0.0356 (11)	0.0030 (9)	0.0093 (9)	−0.0061 (9)
C4	0.0457 (11)	0.0278 (8)	0.0302 (10)	−0.0086 (8)	0.0057 (8)	−0.0008 (8)
C5	0.0348 (9)	0.0270 (9)	0.0235 (9)	−0.0044 (7)	−0.0003 (7)	0.0029 (7)
C6	0.0579 (13)	0.0387 (11)	0.0253 (10)	−0.0166 (10)	−0.0015 (9)	−0.0029 (8)
C7	0.0504 (12)	0.0458 (12)	0.0340 (12)	−0.0204 (10)	−0.0078 (9)	0.0041 (9)
C8	0.0347 (9)	0.0427 (11)	0.0340 (11)	−0.0090 (8)	−0.0040 (8)	0.0107 (8)
C9	0.0325 (9)	0.0317 (9)	0.0275 (10)	−0.0034 (7)	0.0032 (7)	0.0062 (7)
C10	0.0369 (11)	0.0632 (15)	0.0507 (14)	−0.0041 (10)	−0.0093 (10)	0.0208 (12)
C11	0.0378 (11)	0.0656 (15)	0.0572 (15)	0.0163 (11)	0.0031 (10)	0.0239 (13)
C12	0.0486 (12)	0.0514 (13)	0.0380 (12)	0.0166 (10)	0.0035 (9)	0.0067 (10)
C13	0.0377 (9)	0.0342 (10)	0.0283 (10)	−0.0010 (8)	0.0009 (8)	−0.0019 (8)
C14	0.0403 (11)	0.0357 (10)	0.0468 (13)	0.0004 (9)	0.0011 (9)	−0.0078 (9)
C15	0.0364 (14)	0.0334 (15)	0.056 (2)	0.000	−0.0102 (13)	0.000
N1	0.0323 (8)	0.0298 (8)	0.0250 (8)	0.0014 (6)	0.0021 (6)	0.0004 (6)
N2	0.0361 (8)	0.0388 (9)	0.0307 (9)	0.0075 (7)	0.0039 (7)	0.0040 (7)
O1	0.0567 (9)	0.0360 (7)	0.0320 (8)	−0.0025 (6)	0.0138 (6)	0.0001 (6)
O2	0.0795 (12)	0.0528 (10)	0.0653 (12)	−0.0019 (9)	0.0307 (10)	−0.0207 (9)
O3	0.0662 (16)	0.0286 (11)	0.0731 (18)	0.000	−0.0109 (12)	0.000
Zn1	0.0409 (2)	0.03008 (19)	0.0249 (2)	0.000	0.00062 (12)	0.000

### Geometric parameters (Å, °)

**Table:**

C1—N1	1.324 (2)	C10—H10	0.9300
C1—C2	1.391 (3)	C11—C12	1.397 (3)
C1—H1	0.9300	C11—H11	0.9300
C2—C3	1.355 (3)	C12—N2	1.333 (3)
C2—H2	0.9300	C12—H12	0.9300
C3—C4	1.405 (3)	C13—O1	1.277 (2)
C3—H3	0.9300	C13—C13^i^	1.420 (4)
C4—C5	1.412 (3)	C13—C14	1.462 (3)
C4—C6	1.424 (3)	C14—O2	1.229 (3)
C5—N1	1.358 (2)	C14—C15	1.498 (3)
C5—C9	1.433 (3)	C15—O3	1.229 (4)
C6—C7	1.351 (3)	C15—C14^i^	1.498 (3)
C6—H6	0.9300	N1—Zn1	2.1493 (15)
C7—C8	1.430 (3)	N2—Zn1	2.1664 (17)
C7—H7	0.9300	O1—Zn1	2.1325 (14)
C8—C10	1.400 (3)	Zn1—O1^i^	2.1325 (14)
C8—C9	1.406 (3)	Zn1—N1^i^	2.1493 (15)
C9—N2	1.357 (2)	Zn1—N2^i^	2.1664 (17)
C10—C11	1.359 (4)		
			
N1—C1—C2	123.14 (19)	N2—C12—H12	119.0
N1—C1—H1	118.4	C11—C12—H12	119.0
C2—C1—H1	118.4	O1—C13—C13^i^	122.05 (11)
C3—C2—C1	118.91 (19)	O1—C13—C14	127.84 (18)
C3—C2—H2	120.5	C13^i^—C13—C14	110.10 (12)
C1—C2—H2	120.5	O2—C14—C13	127.8 (2)
C2—C3—C4	120.63 (19)	O2—C14—C15	126.6 (2)
C2—C3—H3	119.7	C13—C14—C15	105.61 (19)
C4—C3—H3	119.7	O3—C15—C14^i^	125.84 (13)
C3—C4—C5	116.54 (18)	O3—C15—C14	125.84 (13)
C3—C4—C6	124.48 (19)	C14^i^—C15—C14	108.3 (3)
C5—C4—C6	118.96 (18)	C1—N1—C5	118.26 (17)
N1—C5—C4	122.48 (17)	C1—N1—Zn1	128.09 (13)
N1—C5—C9	118.00 (17)	C5—N1—Zn1	113.65 (12)
C4—C5—C9	119.52 (17)	C12—N2—C9	118.54 (18)
C7—C6—C4	121.43 (19)	C12—N2—Zn1	128.01 (15)
C7—C6—H6	119.3	C9—N2—Zn1	113.37 (12)
C4—C6—H6	119.3	C13—O1—Zn1	107.30 (12)
C6—C7—C8	121.09 (19)	O1—Zn1—O1^i^	81.23 (7)
C6—C7—H7	119.5	O1—Zn1—N1	170.49 (6)
C8—C7—H7	119.5	O1^i^—Zn1—N1	94.20 (5)
C10—C8—C9	117.4 (2)	O1—Zn1—N1^i^	94.20 (5)
C10—C8—C7	123.7 (2)	O1^i^—Zn1—N1^i^	170.49 (6)
C9—C8—C7	118.88 (19)	N1—Zn1—N1^i^	91.48 (8)
N2—C9—C8	122.48 (18)	O1—Zn1—N2	94.54 (6)
N2—C9—C5	117.50 (17)	O1^i^—Zn1—N2	93.84 (6)
C8—C9—C5	120.02 (18)	N1—Zn1—N2	77.37 (6)
C11—C10—C8	119.6 (2)	N1^i^—Zn1—N2	94.83 (6)
C11—C10—H10	120.2	O1—Zn1—N2^i^	93.84 (6)
C8—C10—H10	120.2	O1^i^—Zn1—N2^i^	94.54 (6)
C10—C11—C12	119.9 (2)	N1—Zn1—N2^i^	94.83 (6)
C10—C11—H11	120.0	N1^i^—Zn1—N2^i^	77.37 (6)
C12—C11—H11	120.0	N2—Zn1—N2^i^	168.95 (9)
N2—C12—C11	121.9 (2)		

Symmetry codes: (i) −*x*+2, *y*, −*z*+3/2.

### Hydrogen-bond geometry (Å, °)

**Table:**

*D*—H···*A*	*D*—H	H···*A*	*D*···*A*	*D*—H···*A*
C6—H6···O1^ii^	0.93	2.47	3.295 (2)	149
C11—H11···O3^iii^	0.93	2.55	3.147 (2)	122

Symmetry codes: (ii) *x*, −*y*, *z*−1/2; (iii) *x*−1/2, *y*−1/2, −*z*+3/2.
